# *Actinomycetes*: A Never-Ending Source of Bioactive Compounds—An Overview on Antibiotics Production

**DOI:** 10.3390/antibiotics10050483

**Published:** 2021-04-22

**Authors:** Davide De Simeis, Stefano Serra

**Affiliations:** Consiglio Nazionale delle Ricerche (C.N.R.), Istituto di Scienze e Tecnologie Chimiche, Via Mancinelli 7, 20131 Milano, Italy; dav.biotec01@gmail.com

**Keywords:** antibiotics, *Actinomycetes*, antibiotic resistance, natural products, chemical tailoring, chemical synthesis

## Abstract

The discovery of penicillin by Sir Alexander Fleming in 1928 provided us with access to a new class of compounds useful at fighting bacterial infections: antibiotics. Ever since, a number of studies were carried out to find new molecules with the same activity. Microorganisms belonging to Actinobacteria phylum, the *Actinomycetes*, were the most important sources of antibiotics. Bioactive compounds isolated from this order were also an important inspiration reservoir for pharmaceutical chemists who realized the synthesis of new molecules with antibiotic activity. According to the World Health Organization (WHO), antibiotic resistance is currently one of the biggest threats to global health, food security, and development. The world urgently needs to adopt measures to reduce this risk by finding new antibiotics and changing the way they are used. In this review, we describe the primary role of *Actinomycetes* in the history of antibiotics. Antibiotics produced by these microorganisms, their bioactivities, and how their chemical structures have inspired generations of scientists working in the synthesis of new drugs are described thoroughly.

## 1. Introduction

Antibiotics are a class of molecules used for the treatment and prevention of bacterial infections. It is worth nothing that, before their introduction in clinical practice, there was no effective therapy for the treatment of different medical cases, such as pneumonia, typhoid fever, or gonorrhea [1].

The first natural antibiotic isolated was penicillin, in 1928, by Fleming and co-workers. The scientists, returning from holiday, found one of their Petri-dish containing colonies of *Staphylococcus* contaminated with a mold, later identified as *Penicillium notatum*. Fleming was surprised by the fact that no bacteria were able to growth around the mold; he was, at the same time, perceptive enough to consider the event remarkable.

Despite Fleming’s initial observation, he dealt with chemical issues, such as stability and purification of the bioactive compound. After many years, in 1940, he finally left the idea to isolate the antibiotic substance produced by the mold. Luckily, an Oxford team, guided by Howard Florey and Ernest Chain, managed to purify penicillin in quantities useful for clinical testing. Many firms, such as Lederle, Merck, Pfizer, Squibb, and Abbott Laboratories contributed to solve the concerns related to the scale-up of submerged fermentation. Finally, in 1945, pharmaceutical companies started distributing and trading penicillin [2].

From that moment, pharmaceutical companies and universities began studies in this field, and identified several new bioactive molecules. The antibiotics era started, and its “gold period” was between the 1950s and 1970s, an epoch in which the most known important classes of antibiotics were discovered [1]. These bioactive compounds are produced naturally from different species of fungi and bacteria, but the most attractive class of microorganisms that are able to produce these secondary metabolites are Actinobacteria, in particular, *Actinomycetes*. The importance of this order is due to their abilities to produce different classes of antibiotics in terms of chemical structure and mechanisms of action. Current research suggests that *Actinomycetes* are also a prime resource in the finding of new natural products, thanks to their unique enzymatic sets that permit generating compounds that are potentially useful for diverse purposes. Moreover, different genera and species of *Actinomycetes* are able to produce the same class of antibiotics and, in few cases, the same chemical compound, indicating that the metabolic paths are strictly conserved among the order [3].

Thanks to antibiotics and the research developed in this field, many infections are now treatable, and life-quality/life expectancy are better than the past.

Nature’s beauty is also found in the complexity of evolution, the engine that moves life in order to adapt itself to environmental changes. The fundamental unit of evolution is the mutation, or rather, the change, in the genetic material and the process through which the variation happens.

Through mutations, bacteria become antibiotic-resistant and the infections they cause are harder to eradicate. According to WHO, at least 700,000 people die each year due to drug-resistant diseases, including 230,000 people who die from multidrug-resistant tuberculosis. Several diseases associated with the urinary and respiratory tracts, or sexually transmitted infections, are becoming untreatable. Lower respiratory infections remained the deadliest communicable disease, causing 3.0 million deaths worldwide, especially in low-income countries.

Different causes have contributed to antibiotic-resistance crisis, including factors related to their overuse, inappropriate prescribing, and extensive use in agriculture and breeding [4,5,6].

The WHO and other organizations, such as the Centers for Disease Control and Prevention (CDC), have developed different programs for reducing this phenomenon, with the goal of potentiating the diagnosis and optimizing therapeutic regimens, in addition to adopting a prevention policy, and control tracking and prescribing practices [7].

Recently, scientists shed new light on another interesting topic: the “abuse” of antibiotics in clinics. So far, it seems that abuse of these drugs could cause resistance in commensal bacteria flora. These bacteria are regarded as potential threats. Indeed, if spread out in the environment, they could become vectors for antibiotic resistance in pathogens or could create pathologies in immunocompromised people [8]. In this context, the great diversity of the metabolites with antibiotic activity produced by the Actinomyces is an important tool to tackle the antibiotic-resistance crisis.

This review guides the reader through the different antibiotics produced by microorganisms belonging to the *Actinomycetes* order. These natural products will be described in detail, taking into consideration their peculiarities, in terms of both bioactivity and chemical structures. Further topics that will be examined concern their industrial preparations. In particular, the variability and the complexity of their chemical structures have challenged organic chemists, inspiring researches who are “finalized” to the synthesis of these natural products or to the creation of other useful bioactive compounds.

## 2. *Actinomycetes*: Biology and Bioactive Compounds

*Actinomycetes* are microorganisms belonging to the order of Actinomycetales, whose phylum is Actinobacteria, one of the largest taxonomic units within the bacteria domain. The phylum comprises Gram-positive bacteria with high guanine-cytosine (G + C) content in their DNA, ranging from 51% in some corynebacteria to more than 70% in Streptomyces and Frankia. An exception is the genome of the obligate pathogen *Tropheryma whipplei*, in which the G + C content is less than 50% [9]. The phylum is also adapted to a wide range of environments, such as soil, water (even salty), and air. Nevertheless, the predominant part of these organisms is preferentially found in alkaline soil with big amounts of organic matter [10].

From a morphological point of view, actinobacteria show different features. Indeed, we can recognize microorganisms with a coccoid/rod-coccoid shape-like species belonging to the suborder of Micrococcineae, or with a fragmenting hyphal form (microorganisms belonging to the suborder of the Corynebacterineae, such as *Nocardia* spp.), or with a permanent and highly differentiated branched mycelium, such as *Streptomyces* spp. [11].

**Figure 1:**
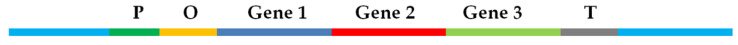
Schematic view of an operon: promoter (P); operator (O); operon gene and terminator (T).

From a genetic point of view, genes involved in the biosynthesis of antibiotics, and other metabolites belonging to the secondary metabolism, are located in the genome of the microorganism, and rarely in plasmids. They are clustered in very long operons, whose general assembling is schematized in Figure 1. Commonly, it is possible to identify codifying sequences (genes) and regulatory sequences (P and O) that are able to promote or inhibit the gene expression responding to specific environmental changings. These genes encode enzymes involved in the assembling and editing of the bioactive compound, in protecting the cell from its toxicity, and facilitating its extrusion in the environment [12,13].

It is worth noting that the production of secondary metabolites in *Actinomycetes* is strictly dependent on cell morphological and physiological differentiation. The factors that influence the microorganism’s behavior is not well understood yet, but reasonably, variations, such as nutrient concentration and accessibility, competitor occurrence in the environment, metabolites, and cellular density, could influence the gene expression, and the enzymatic set inside the cell as a consequence [14,15].

Antibiotics are always conceived as molecules able to kill other bacteria, but their functions are more complicated than we thought. This simplification arose from a laboratory “distortion” in which we considered the antibiotic producer as an isolated system. Indeed, considering a composite environment, in which different microorganism populations coexist, these molecules could have different roles, such as signaling agents, a promoter of the biofilm formation, or, in general, influencing the gene expression of the producer microorganism itself or of the neighbor [16,17,18]. Interestingly, a large number of Actinomycete-derived antibiotics were isolated from microorganisms that complete their whole lifecycles in soils—especially biologically polluted soils. This is further proof that the microbial-production of antibiotic compounds can be an evolutive advantage that allows microbial producers to compete better for natural resources.

Another important point refers to the unique conditions present in a deep marine environment. It is presumed that marine *Actinomycetes* may have peculiar characteristics with unique structural elements that have not previously been found in terrestrial *Actinomycetes*, due the extreme variations in ecological pressure, including competition for space, predation, available nutrients, light, oxygen concentration, and pressure [19,20].

## 3. Mechanisms of Resistance and Antibiotics from *Actinomycetes*: An Overview

The antibiotic activity of a molecule is dependent on its chemical structure and, thus, to its affinity to a specific biological target. From a chemical point of view, there are different antibiotic classes produced by *Actinomycetes*. Indeed, it is possible to identify aminoglycosides, peptides, ansamycins, β-lactams, tetracyclines, macrolides, lincosamides, epoxides, and aminocoumarins.

In regards to biological activity, a bioactive compound, useful for clinical use as antibiotic, must act on a specific prokaryotic target without damage to the eukaryotic system. There are many opportunities in this sense because the bacteria biology and morphology are quite different from those of the eukaryotic cell.

Currently, the most important antibiotic targets refer to the cell wall biosynthesis, to the DNA wrapping, to the translation or transcription steps, and to some specific traits of the bacterial metabolism.

**Figure 2:**
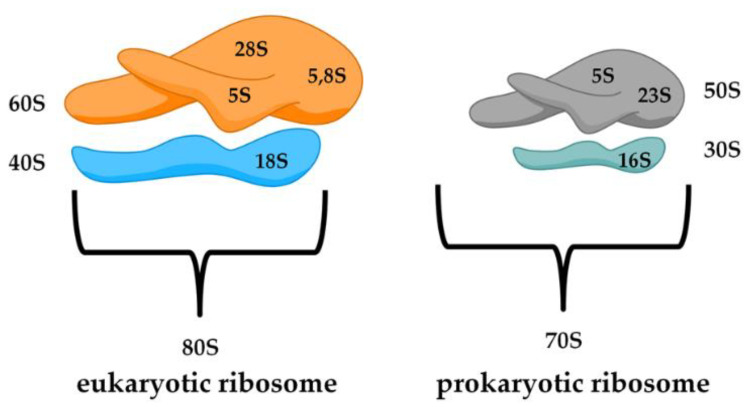
Schematic representation of a eukaryotic and prokaryotic ribosome.

A relevant diversity between the prokaryotic and eukaryotic biological structure lies in the ribosome. Bacterial ribosomes are smaller (70S) with respect to the eukaryotic ones (80S) and consist of two subunits, 50S and 30S (Figure 2). Moreover, the small subunit 30S is composed of rRNA 16S, while the eukaryotic subunit 40S, is formed by rRNA 18S. It is possible to notice differences in the major subunit of the ribosome because bacteria (50S) is composed of rRNA 5S and 23S, while in eukarya (60S) we find 28S, 5.8S, and 5S. Another important difference is in the protein content of the ribosome. The RNA percentage in bacteria is higher than the protein part (60% rRNA and 40% peptides), while in the eukaryotic cells, the ratio is completely inverted [21].

The most common antibiotics active in bacterial protein synthesis are selective for the ribosome RNA, and they belong to different chemical classes, such as aminoglycosides, tetracyclines, and macrolides [22]. The capability to discriminate between a eukaryotic ribosome and one belonging to bacteria depends on many aspects. Nevertheless, the most important seems to be attributed to RNA sequence. Many studies demonstrated that, in many sensitive bacteria that became resistant to the drug, the nucleotides responsible for the antibiotic binding site were changed [23].

In regards to microbial metabolism, there are specific biosynthetic pathways only identified in prokaryotic systems that could be considered an interesting target for novel drugs to use as antibiotics. One historical example is related to folic acid biosynthesis. Indeed, folic acid is indispensable for both humans and infecting pathogens. However, humans obtain this substance as a vitamin in the diet, and do not need a de novo biosynthetic pathway for its synthesis. On the contrary, bacterial cell walls are impermeable to folic acid and its derivatives, because they lack the necessary folate receptors that internalize these substances. These considerations drove scientists to develop drugs that were able to interfere with this process, and different products were commercialized, such as sulfadiazine and sulfacetamide [24].

It is worth noting that many effective drugs act via modulation of multiple essential proteins rather than a single one because the likelihood of two simultaneous mutations occurring in two essential targets is low and limited [25]. Recently, scientists considered the possibility of “cut down” virulence in pathogens, even though they are nonessential to the survival. This idea was born, postulating a low-rate of mutation and a low possibility of developing resistance mechanisms due to little, selective pressure derived from their loss. Nevertheless, different studies suggested the onset of resistance mechanisms even for these nonessential targets [26].

There are several resistance mechanisms in bacteria that could be summarized as follows:

1. Permeability alteration;
2. Target modification/amplification;
3. Drug inactivation.

The susceptibility of the bacterial pathogens to antibiotics is strictly related to membranes permeability. Small hydrophilic drugs, such as β-lactams and fluoroquinolones, use pore-forming porins to gain access to the cell interior, while macrolides and other larger hydrophobic drugs diffuse across the lipid bi-layers. The existence of drug-resistant strains in a large number of bacterial species is due to the modification of membrane components involved in passive diffusion and active transport (influx and efflux) in terms of presence, production levels, and structures [27].

Another important resistance mechanism involves target modification/amplification. The alteration of the antibiotic target as a result of mutation, chemical modification, substitution, or masking of key binding elements, makes the drug useless for therapeutic use. The same effect could be reached by gene duplication/amplification of the antibiotic target.

Several mechanisms were suggested that could increase gene copy number; considering the duplication, it can occur following multiple mechanisms, such as non-equal homologous recombination between directly orientated repeats, RecA-independent mechanisms, and through rolling circle replication [28].

In case of target amplification, the antibiotic is still able to perform its activity, but a higher concentration is required to exploit the effect. Therefore, the clinical treatment could become incompatible due to the inherent toxicity of the drug.

Drug inactivation is the last mechanism through which bacteria are able to alter the chemical structure of antibiotics, making them inactive. Different research groups have recognized a number of enzymatic inactivation processes, which involve the most important antibiotic classes. These chemical transformations comprise hydrolysis, group transfer, and redox (mainly oxidation) reactions [29,30,31,32].

### 3.1. β-Lactam Antibiotics

The prokaryotic cell wall is composed of different proteins, lipids, and sugars, organized in particular manners depending on the species analyzed. Peptidoglycan is a particular biological structure, present predominantly in the Gram-positive bacteria wall, made from polysaccharide chains consisting of *N*-acetylglucosamine (NAG) and *N*-acetylmuramic acid (NAM), cross-linked together by short peptides containing modified amino acids, such as aminopimelic acid (DAP) and native L-or D-amino acids. From a functional point of view, the bacterial cell wall is the most important structure that permits bacteria to maintain its shape and respond efficiently to environmental stresses, maintaining the osmotic balance [33].

**Figure 3:**
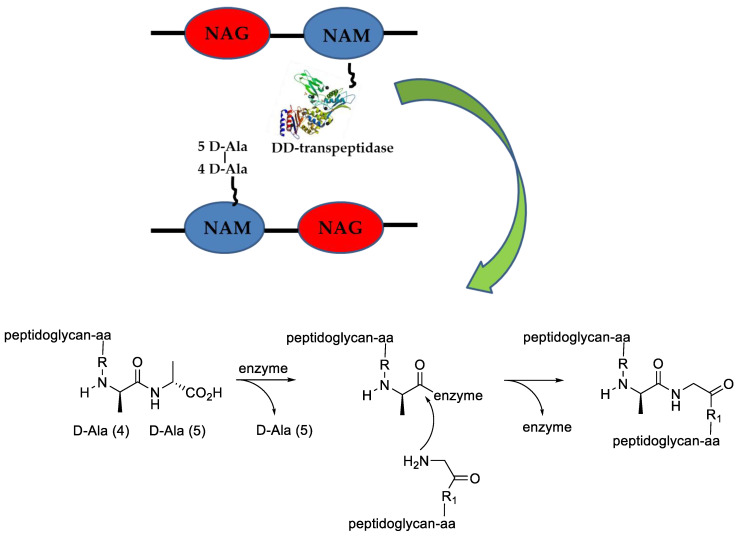
Peptidoglycan cross-linking mediated by DD-transpeptidase. The “serpentine” attached to NAM in black represents the peptide chain. The enzyme image is kindly taken by PDB [36].

In coccoid bacteria, the peptidoglycan biosynthesis starts in the cytoplasm, where NAM is linked with a pentapeptide that contains, far from the sugar moiety, two D-Alanine amino acids (D-Ala-D-Ala). The macromolecule is linked to the inner part of the cell membrane where the NAG and other amino acids are added. This intermediate is now carried out from the cell where other units of the same monomer are added via transglycosylation. Different, growing polymer strands are then cross-linked by the action of a class of enzymes called transpeptidase, able to recognize and link diverse peptidoglycan regions through the D-Ala-D-Ala moiety (Figure 3). It is worth noting that the connection formed by transpeptidase is dependent on the species; some examples are well described in the literature [34,35].

β-Lactams antibiotics are molecules produced from bacterial and fungal species, with (generally) bactericidal activity. Their biological actions are correlated to the inhibition of the cell wall biosynthesis, resulting from the irreversible transpeptidase active site block due to the similarity of the antibiotic with the D-Ala-D-Ala moiety described above.

**Figure 4:**
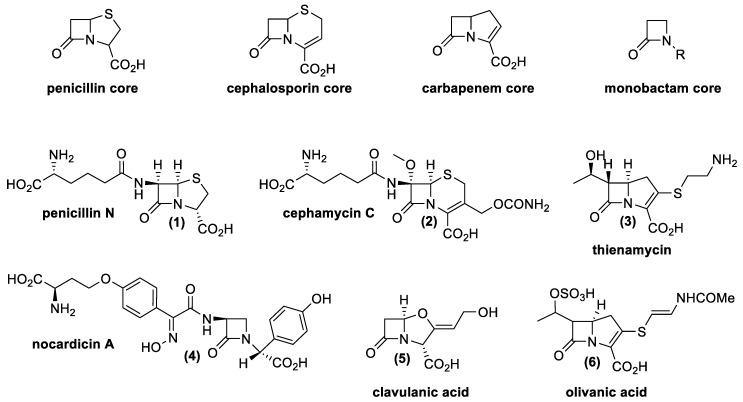
Base structural nucleus of β-lactam antibiotics and some representative examples of natural beta-lactam antibiotics produced by *Actinomycetes* [38,39,40,41,42,43,44,45,46,47,48,49,50,51].

From a chemical point of view, there are four main classes of natural β-lactams, all of them containing a four-membered cyclic amide (Figure 4).

Penicillin possesses a β-lactam ring, fused with a five-membered ring with a sulfur atom; cephalosporins possess a β-lactam ring with a six-membered unsaturated ring with a sulfur atom, and carbapenems possess an unsaturated five-membered ring without heteroatom. Finally, monobactams only contain the β-lactam ring [37].

Resistance mechanisms that are generally recognized refer to the production of efflux pumps; modification of the antibiotic target; and synthesis of enzymes able to inactivate the antibiotic (β-lactamase, serine or Zn^2+^ proteases). Sometimes the latter problem could be avoided thanks to a particular class of β-lactam antibiotics called β-lactamase inhibitors. Clavulanic acid (**5**) and olivanic acid (**6**) are two natural molecules belonging to this group of antibiotics that work as suicide inhibitors of these particular enzymes. The discovery of these drugs and their natural producers had a big significance from a scientific and pharmaceutical point of view. Indeed, the finding has permitted the development of combined therapies using β-lactams and β-lactamase inhibitors, even against bacteria resistant to this antibiotic class. In nature, many compounds have these characteristics, produced by different *Actinomycetes* as well as fungal species. Table 1 presents natural β-lactam antibiotics and β-lactamase inhibitors produced by *Actinomycetes*.

### 3.2. Ansamycines

**Figure 5:**
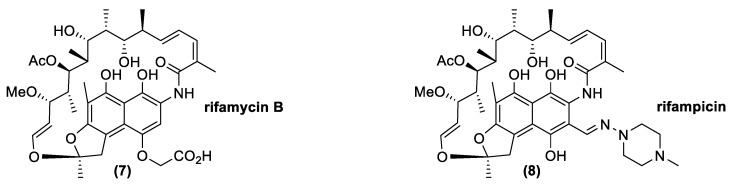
Natural and synthetic ansamycins: rifamycin B (natural) and rifampicin (semisynthetic).

Ansamycins are naturally occurring compounds produced by diverse microorganisms as secondary metabolites. Chemically, they contain aromatic moieties bridged by a polyketide chain terminating in an amide connection (Figure 5). The most important natural antibiotic with this kind of structure is rifamycin B (**7**). Clinically, rifamycin B does not have a primary role because it has low biological activity. Nevertheless, its production is very important since it is easy to produce by fermentation and it is simple to convert in other derivatives, such as rifamycin S, an important building block for the synthesis of other drugs, such as rifampicin (**8**), which is widely exploited in clinical practices. Attention on this antibiotic class depends, above all, on its activity against *Mycobacterium*, but it is also generally important for treatment of other pathogenic Gram-positive bacteria, such as *Staphylococcus aureus* and *Streptococcus* spp. [52].

Rifamycins are antibiotics with bactericidal activity, due to their ability to inhibit DNA-dependent RNA polymerase. The most common resistance mechanism is due to the modification of the antibiotic target through a chromosomic mutation of the DNA-dependent RNA polymerase. The mutation rate is very high when rifamycin is used in monotherapy and, for this reason, it is always associated with a second antibiotic to reduce this issue [53].

Rifamycin derivatives are produced by different microorganisms belonging to the *Actinomycetes* group, such as *Actinomadura formosensis*, *Amycolatopsis tolypomycina*, *Micromonospora* sp., *Amycolatopsis mediterranei*, *Amycolatopsis rifamycinica* [38,39], *Micromonospora lacustris* [54], and *Salinispora arenicola* [55].

### 3.3. Macrolides

The natural macrolides class encloses a series of compounds with 14, 15 or 16 carbon atoms organized as a macrocyclic lactone, in which are often present one or two sugar moieties as “ring decorations”. The differences among these molecules are related to their pharmacokinetic properties, tolerability, and metabolization [56]. Macrolides are generally considered bacteriostatic agents but, in the same cases, they could reveal bactericidal activity. From a molecular point of view, they interact with the 50S subunit of the prokaryotic ribosome, inhibiting the translocation of the ribosome. The mechanisms of resistance are disparate and cross-resistance to other antibiotics with similar structures (lincosamides and streptogramins) are often associated. Generally, it is possible to observe resistances related to efflux pumps and modification of the target (70–100% of the clinical isolate). Strains able to inactivate the antibiotic are rarely found [57,58].

In nature, macrolides were isolated from different *Streptomyces* spp.

**Figure 6:**
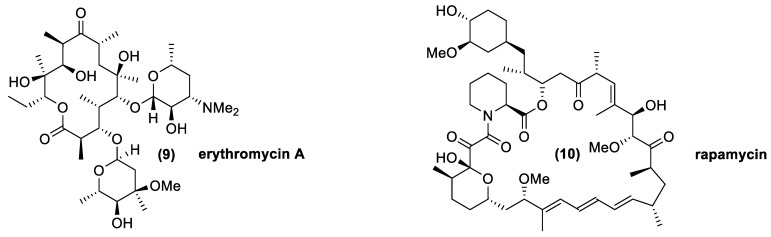
Macrolide molecules produce by *Actinomycetes*: erythromycin A (antibiotic), and rapamycin (immunosuppressant).

The most common macrolides are oleandomycin, isolated from *Streptomyces antibioticus*; spiramycin, from *Streptomyces ambofaciens*; josamycin, from *Streptomyces narbonensis* var. *josamyceticus*; midecamycin, from *Streptomyces mycarofaciens*; rosaramycin, from *Micromonospora rosaria*; and erythromycin, isolated from *Aeromicrobium erythreum* and *Streptomyces erythreus* [38,39,59,60,61,62,63]. The last mentioned compound (**9**), shown in Figure 6, is the most important and widely-used macrolide molecule, even if the others, excluding rosaramycin, which was eliminated for its neurotoxicity, are also used as medical agents thanks their therapeutic and bioactive features. Other macrolides, such as lipiarmycin and rapamycin (**10**), possess larger lactone rings. Interestingly, the latter macrolide was isolated from cultures of *Streptomyces hygroscopicus* collected from Easter Island. This compound was named rapamycin, according to the native name of the island, Rapa Nui, and was initially used as an antifungal agent. The discovery of its immunosuppressant properties made it the drug of choice for the prevention of organ transplantation rejection. This is a further example of the great diversity of the metabolites produced by *Streptomyces* strains, both in terms of chemical structure and bioactivity [64].

### 3.4. Lincosamides

**Figure 7:**
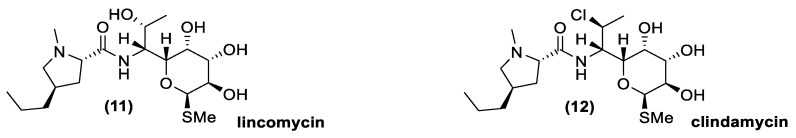
Natural and synthetic lincosamides: lincomycin (natural) and clindamycin (semisynthetic).

Lincosamides are very similar to macrolides in terms of activity spectrum and mechanisms of action, but their chemical structures are quite different (Figure 7). Indeed, no macrocyclic lactone is present and the sugar moiety is attached to an amino acid element. The resistance mechanisms are often linked to those of macrolides and streptogramins, in which a methylase induces an alteration on the 50S subunit of the ribosome. In nature, lincomycin (**11**) was the first lincosamide isolate, but its semisynthetic chlorine derivate clindamycin (**12**) is usually more active than lincomycin in the treatment of bacterial infections, in particular those caused by anaerobic species [65]. Lincomycin is available from different *Actinomycetes* species, such as *Streptomyces lincolnensis*, *Streptomyces spinosus*, *Streptomyces pseudogriseolus*, *Streptomyces variabilis*, *Streptomyces variabilis var. liniabilis*, *Streptomyces vellosus*, *Micromonospora halophytica*, and *Actinomyces roseolus* [38,39,66,67,68,69,70,71].

### 3.5. Tetracyclines

**Figure 8:**
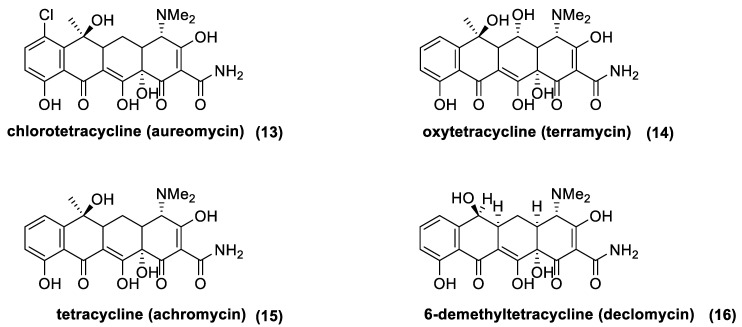
Natural tetracycline: chlortetracycline (**13**), oxytetracycline (**14**), tetracycline (**15**), and 6-demethyltetracycline (**16**).

Tetracyclines are a class of antibiotic compounds discovered in the second half of 20th century. Chemically, they possess a naphthacene carboxamide nucleus generally substituted on carbon C2, C4, C6, and C7 of the naphthacene core. These molecules show a bacteriostatic effect inhibiting the protein synthesis through the binding with the 30S subunit of the prokaryotic ribosome. Different resistance mechanisms were identified in plasmid or transposon DNA. These genes confer to the bacteria the ability to extrude the antibiotic from the cell through efflux pump (generally in Gram-negative bacteria) or to modify structurally the ribosome in order to hinder the binding with the antibiotic (in the main part of the Gram-positive bacteria). Being that the resistance is usually located in “mobile-DNA”, the activity spectrum of tetracycline has become tighter with respect to its initial action radius. In fact, first generation tetracycline is recommended only for the treatment of intracellular pathogens, such as *Brucella*, *Rickettsia*, or *Chlamydia*. The first natural tetracycline approved for clinical use by the FDA was chlortetracycline (**13**) (1948) followed by oxytetracycline (**14**) (1951), tetracycline (**15**) (1953), and demethylchlortetracycline (**16**) [72,73] (Figure 8). These compounds are produced by many *Actinomycetes* species, which are summarized in Table 2 (first generation tetracyclines).

### 3.6. Aminoglycosides

Aminoglycosides are antibiotic drugs generally formed by two or three amino sugars linked with a glycosidic bond to a central aminocyclitol core. Moreover, thanks to the amino groups in the molecule, the aminoglycosides possess a polycationic character at physiological pH. These antibiotics present different issues linked to their oto- and nephrotoxicity, but also to their narrow therapeutic range. Nevertheless, they are considered fundamental and are often associated with β-lactams for the treatment of rigid infectious thanks to their capability of rapidly killing bacteria and for their post-antibiotic effects [78].

Due their cationic properties, aminoglycosides interact almost instantaneously with the anionic spots in the cell surfaces. The kinetic is dependent on concentration and the interaction is reversible. It is worth noting that other cationic species could influence this process negatively.

The mechanisms through which aminoglycosides reach the cytoplasm are several, and among them, it is possible to highlight the active transport; the facilitate transport, (strictly related to the membrane potential and antibiotic concentration); and the self-promoted uptake pathway (common in Gram-negative bacteria), in which aminoglycosides alter the structure of the cross-bridged lipopolysaccharide molecules (LPS) in the outer membrane. This latter mechanism allows the generation of pores that permit a better cell-permeability to aminoglycosides [79,80,81].

Their most important activity consists in the alteration of the protein synthesis due to their binding on the 16S rRNA of the ribosome. The interaction with ribosomes causes an aberrant protein synthesis in which truncated proteins or proteins with an altered sequence are produced. Moreover, the aminoglycoside-binding also impairs the ribosome translocation [73,81,82]. The binding specificity of these compounds is only 10-fold higher in favor of the prokaryotic ribosome and this detail could explain the toxicity for the mammalian cell. Although the major actions of aminoglycosides are carried out through the ribosome impairing, the major resistance mechanisms move through the expression of specific enzymes able to modify the aminoglycoside antibiotic and by the reduction of the antibiotic permeability inside the cell. These processes are in part due to the evolutionary stability of the ribosome that remains conserved among the species, and because microorganisms possess multiple gene copies codifying for rRNA [82]. It is important to underline that a ribosomal resistance exists and was noticed for example in a clinical isolate of the *E. coli* strain ARS3, and other strains distributed worldwide belonging to *Enterobacteriaceae*, but is very rarely, with respect to the others, cited above [83,84].

**Figure 9:**
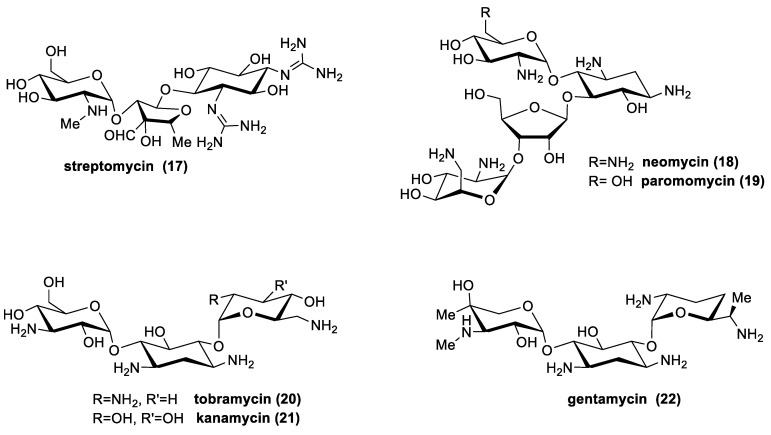
Selected examples of natural aminoglycosides: streptomycin, neomycin, paromomycin, tobramycin, kanamycin and gentamycin [38,39,53,85,86,87,88,89,90,91].

There are many aminoglycosides produced by *Actinomycetes* in nature (Figure 9), and are generally biosynthesized by *streptomyces* spp. (streptomycin; kanamycin; neomycin; tobramycin; paromomycin and spectinomycin); or by *Micromonospora* spp. (gentamicin; sisomicin; netilmicin; sagamicin; fortimicin; and dactimicin) [53].

The major compounds used and their microbiological sources are presented in Table 3.

### 3.7. Antibiotic Peptides

Antibiotic peptides is a (very interesting) class of compounds from a biosynthetic point of view. Indeed, it is possible to distinguish two groups in which these molecules could be gathered: the ribosomal peptide synthetases and the nonribosomal peptide synthetases (NRPSs). However, our interest is focused on NRPSs because the major part of antibiotics produced by *Actinomycetes* spp. (lipopeptide, glycopeptides, and streptogramins) is related to this mechanism [92,93,94,95].

**Figure 10:**
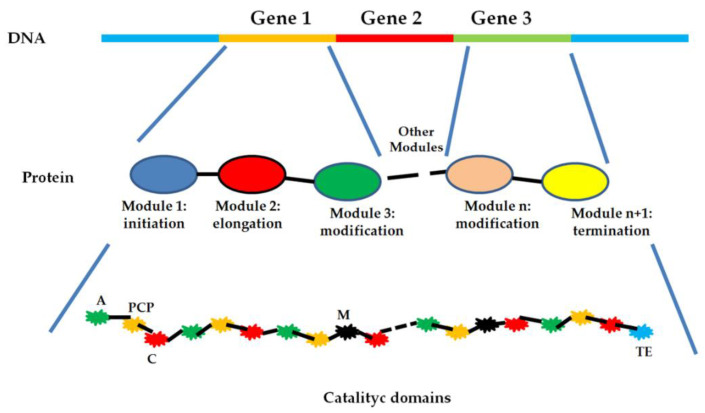
Schematic representation of an NRPS enzyme. A gene codifies for a module; each module is specific for the sequence and for the final structure of the peptidic product; each module possess catalytic domains: the main are the activation domain (**green**, A), carrier protein (**yellow**, PCP), condensation domain (**red**, C), modification domain (**black**, E), and termination domain (**light blue**, TE).

NRPSs are assembled on multi-modular enzymes that are able to generate a macrocyclic structure acting as a protein template. These multi-modular proteins are called nonribosomal peptide synthetases (NRPSs). It is important to underline that these particular enzymes are able to synthetize peptides, accepting as building blocks, not only the 20 classic proteinogenic amino acids, but also lipids, α-hydroxy acids, and non-proteinogenic amino acids. Although the different enzymes catalyze a specific synthetic step, the chemical and sequence identity of the product is strictly dependent on the order in which the multi-modular enzyme is assembled. In each NRPS module, it is possible to identify different catalytic domains responsible for the synthesis of the final product (Figure 10). Usually, there is a domain responsible for the amino acid activation (adenylation domains, (**A**) a carrier unit implied in the movement of the growing peptide in the different regions of the NRPSs (**PCP**), a condensation domain, useful for the amide-bond formation (**C**), and a thioesterase domain (**TE**) able to catalyze the hydrolysis or the macrocyclization of the product. Often, it is easy to find other catalytic domains responsible for the structural modification of the final product (**M**), such as epimerization, methylation, or cyclization. These domains are inserted in different positions of the various modules that constitute the NRPSs. Moreover, other independent enzyme(s) could introduce further modifications using the rising-product as substrate [95,96,97,98].

**Figure 11:**
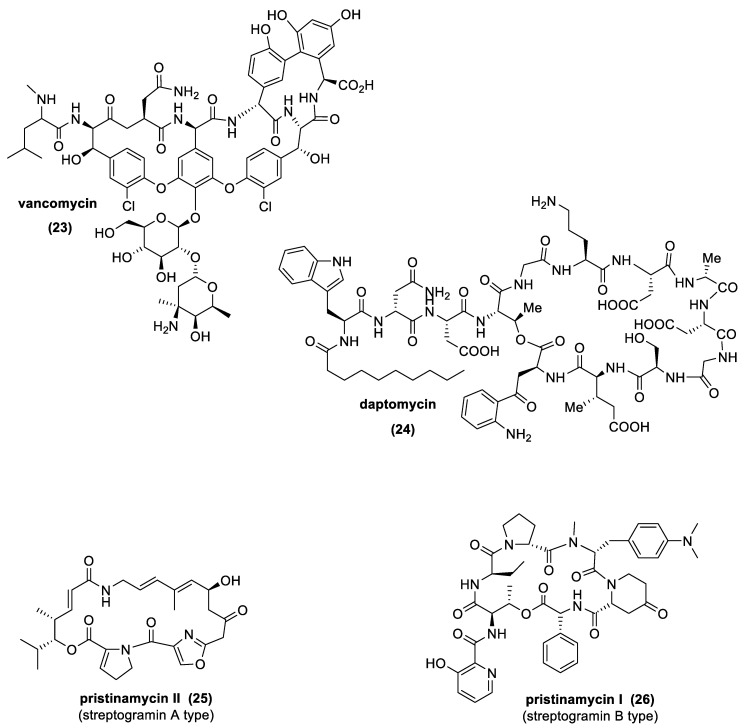
Selected examples of natural peptide antibiotics produced by *Actinomycetes*: vancomycin, daptomycin, pristinamycin II (streptogramin A type), and pristinamycin I (streptogramin B type) [38,39,105,106,107,108,109].

The most important natural antibiotics of this class, produced by different *Actinomycetes* spp., are glycopeptides, such as vancomycin (GPAs), lipopeptides, such as daptomycin (LPAs), and streptogramins (Figure 11). From a chemical point of view, GPAs and LPAs are molecules containing a linear or cyclic peptidic scaffold decorated with different sugars (GPAs) or fatty acids (LPAs) and other particular functional groups, such as chlorine, acetyl, or methyl groups. Conversely, streptogramins possess characteristics very close to macrolides, exhibiting a macrocyclic polyunsaturated lactone structure (streptogramins A), while, in the case of streptogramins B, a cyclic hepta-or hexadepsipeptide structure is observed [94,99].

Although LPAs and GPAs are quite similar structurally, they have different mechanisms of action.

GPAs are able to impair the cell wall synthesis blocking the transpeptidation and transglycosylation reactions through the binding with the D-Ala-D-Ala moieties, stabilized by wake–energy bonds, such as hydrophobic van der Waals contacts and hydrogen bonds [100]. LPAs, instead, are able to act as surfactants, binding directly to the bacterial membrane, causing a misbalance of the chemicophysical properties of the same biological structure [101]. The resistance developed against GPA antibiotics are generally related to the capability to substitute the D-Ala-D-Ala moiety with a D-Ala-D-Lactate framework, an alteration that could decrease the interaction with the antibiotic up to 1000-fold. In addition, bacteria can become resistant to GPAs, increasing the thickness of the cell wall (substrate iper-production) [73,102,103].

Contrarily, streptogramins possess the same spectrum of activity and mechanism of action of macrolides, and the resistance mechanisms are often related. Separately, group A and group B streptogramins are bacteriostatic, by reversible binding to the 50S subunit of 70S bacterial ribosomes. Together, however, streptogramins from each group are synergic and bactericidal [104].

Regarding the activity of GPAs and LPAs against pathogens, we can assume they have a similar spectrum of action, especially against Gram-positive bacteria, even against strains resistant to other antibiotics.

The most important antibiotic peptides produced in nature by *Actinomycetes* are summarized in Table 4.

### 3.8. Aminocumarines

**Figure 12:**
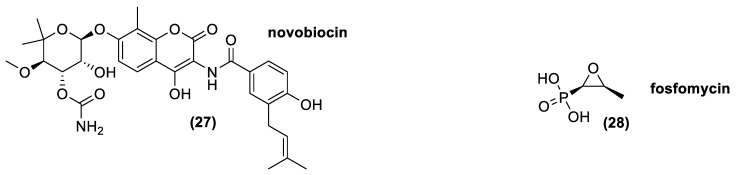
The natural aminocoumarin novobiocin and the epoxide antibiotic fosfomycin.

Aminocoumarins is an antibiotic class chemically formed by a 3-amino-4,7-dihydroxycumarin ring diversely decorated in its different position. Novobiocin (**20**) is the most important natural molecule belonging to this class, which was approved for animal uses (Figure 12). Indeed, Novobiocin, previously employed for human use under the name albamycin, was withdrawn from the market by the FDA due to lack of safety or effectiveness [110].

Novobiocin is composed by a sugar residue (novobiose) and a benzoic acid derivate attached to the coumarin ring. The major novobiocin producers, *Actinomycetes*, are *Streptomyces spheroides*, *Streptomyces caeruleus*, and *Streptomyces niveus* [38,39,111], even though *Streptomyces spheroides* could be considered as a heterotypic synonym of *Streptomyces niveus* [112]. Aminocoumarins are able to inhibit DNA gyrase, an enzyme essential for making accessible the DNA to all the enzymes implied in replication and translation. Aminocoumarins bind with the β-subunit of the heterotrimeric enzyme, partly covering the ATP-binding site of the gyrase hindering, the ATP hydrolysis required for ATP-dependent DNA supercoiling. Moreover, an activity of aminocoumarins against DNA topoisomerase IV, an enzyme also involved in the control of DNA supercoiling and in the decatenation of the daughter chromosomes after DNA replication in bacteria, was shown [113,114].

Novobiocin is a narrow-spectrum antibiotic that may be bacteriostatic or bactericidal at higher concentrations. It is active mostly against Gram-positive bacteria, but also against a few Gram-negative bacteria, including *Neisseria*, *Bordetella*, *Brucella*, *Haemophilus*, and some strain of *Proteus* [115]. The resistance acquired in bacteria are generally related to modification in the antibiotic target, puntiform mutation that impair the binding between the drug and the enzyme [116].

### 3.9. Epoxides

The most important natural molecule belonging to this class is L-*cis*-1,2-epoxypropylphosphonic acid, known as fosfomycin (**28**). This is a polar compound in which the epoxide framework is determinant for its bactericidal activity. It is an antibiotic with a relatively broad activity spectrum because it carries out its function against most Gram+ and Gram- bacteria. It inhibits, irreversibly, the action of MurA, an enzyme involved in the first step of the peptidoglycan synthesis. Fosfomycin is always associated with another antibiotic, because in monotherapy, it often causes the onset of resistant bacteria strains. The major resistance mechanisms are associated with a reduced uptake of fosfomycin due to mutation(s) in the structural genes codifying the transporters, an over-expression or modification of the target enzyme MurA, or through antibiotic inactivation [117,118].

In nature, it is produced by different *Actinomycetes*, such as *Streptomyces fradiae*; *Streptomyces viridochromogenes*; and *Streptomyces wedmorensis* [53,119,120,121,122]. Moreover, thanks to genetic engineering, a strain of *Streptomyces lividans* is now able to produce fosfomycin. Indeed, Woodyer and co-workers in 2006, identify the minimal biosynthetic cluster of fosfomycin in *Streptomyces fradiae*, giving a new tool and the possibility to overproduce the antibiotic and create new useful analogues [123].

## 4. Antibiotics from *Actinomycetes*: From Isolation to Chemical Characterization, Total Synthesis, and Industrial Production

The antibiotics produced by *Actinomycetes* possess the most varied chemical structures, which display a wide range of biological activities. As it happens for the main part of APIs, the structure–activity relationship (SAR) of these molecules was studied thoroughly, in order to develop new and more effective drugs. In this context, the antibiotics isolated from this class of bacteria were a wonderful source of inspiration for generations of chemists who have decided to challenge their skills. Indeed, the high structural complexity of these natural products makes it difficult to determine their chemical structures, as well as the accomplishments of their syntheses or the preparation of new synthetic analogues. It is not surprisingly that different scientists, awarded with the Nobel Prize, spent part of their careers engaging the syntheses of specific antibiotics produced by *Actinomycetes* [124,125].

According to their industrial manufacturing methods, antibiotics can be classified in three separate categories: natural compounds, semisynthetic antibiotics, and fully synthetic antibiotics. Natural antibiotics are obtained directly by large-scale fermentation of a given microbial strain whereas semisynthetic derivatives are compounds manufactured by chemical transformation of a natural product. Fully synthetic antibiotics are prepared exclusively through synthetic routes. Actinomyces were sources of very large numbers of natural and semisynthetic antibiotics and are producers of secondary metabolites whose chemical structures have inspired the discovery of new fully synthetic antibiotics. Each one of the above-described classes of pharmaceutical compounds include relevant drugs, whose histories started thanks to the research on Actinomyces strains. Of course, it is impossible to describe comprehensively all of the chemical efforts that have been spent to study these compounds. Therefore, we reported below only a few relevant examples of the aforementioned antibiotics whose isolations, chemical characterizations, total syntheses, and industrial productions can be regarded as milestones of the pharmacology and pharmaceutical industries.

### 4.1. Penicillin and Cephalosporin

**Figure 13:**
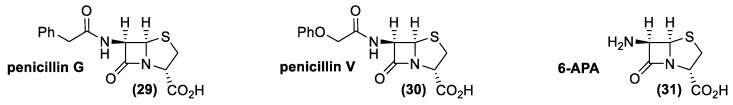
Milestone compounds in penicillin production: penicillin G, penicillin V, and 6-aminopenicillanic acid (6-APA).

Large-scale production of penicillin and cephalosporin were strictly connected to two relevant industrial problems: finding a suitable, high producing strain and the development of antibiotic resistances. Since the isolation of the first penicillin (**29**, penicillin G, 1940), chemists have faced the problem of the synthesis of this important drug (Figure 13). Unfortunately, difficulty in assembling the high reactive beta-lactam structure delayed (for more than 15 years) the first synthesis of a member of this class of compounds (**30**, penicillin V, 1957) [126].

Consequently, the penicillin requirement fostered the quest for a biotechnological method of its production, which finally lead to the selection of suitable high-producing strains. The following development of a reliable fermentative process secured the large-scale production of penicillin G, which quickly found a number of medical applications. Unluckily, many bacterial strains became resistant to the latter antibiotic, which was phased out of clinical use in the late 1960s. In spite of this fact, the discovery of a new class of beta-lactam antibiotics, the cephalosporins (cephalosporin C, 1955) [127], broaden the pharmaceutical relevance of this class of compounds. In addition, some penicillin and cephalosporin antibiotics possessing new chemical structures were isolated from *Actinomycetes* (penicillin N and cephamycins). These findings indicated that only the β-lactam ring is essential for the antibiotic activity and suggested that the chemical modification of the ‘substituents’ linked to the bicyclic moiety could affect the bioactivity of these compounds.

**Figure 14:**
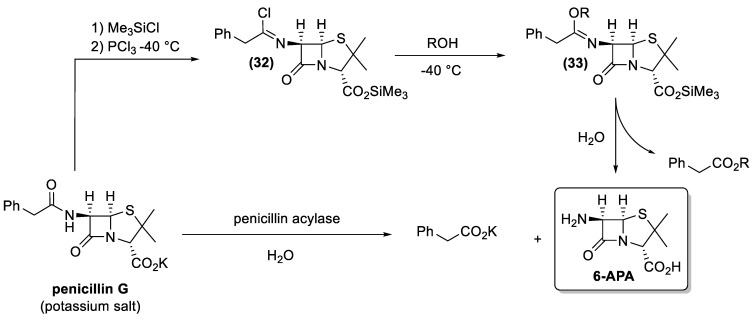
The industrial processes to 6-aminopenicillanic acid (6-APA).

Taking advantage of the already established process for penicillin G production, chemical efforts were then focused on the development of new semi-synthetic antibiotics. From a chemical standpoint, the use of 6-aminopenicillanic acid (**31**, 6-APA) as the starting material for the syntheses of these new derivatives was the most logical choice (Figure 14). Therefore, the easily available penicillin G was used as starting material in 6-APA production. Nevertheless, the cleavage of the phenylacetyl moiety from the latter antibiotic is not an easy transformation. The presence of two amide functional groups makes it necessary for a high regioselective deacylation reaction. In addition, the β-lactam ring is very sensitive to hydrolysis and basic reaction conditions lead to the lactam opening.

To overcome these problems, an ingenious and reliable cleavage procedure was developed in the late 1970s [128]. Accordingly, the carboxylate functional group of the penicillin G salt is protected as trimethylsilyl ester, whereas the secondary amide bond is transformed into an imine chloride, through reaction with phosphorus trichloride at low temperatures (−40 °C). The obtained intermediate (**32**) is treated with an alcohol (usually butanol) to give derivative (**33**), whose quenching with water leads to the final cleavage of the phenylacetic moiety as the corresponding alcohol-ester. The 6-APA is obtained in high overall yield and the above-described process was largely employed in the industry for about 20 years after its discovery.

The chemical methods for producing 6-APA are environmentally burdensome as they require hazardous chemicals and generate a considerable amount of waste. Therefore, the development of new enzymatic processes has received increasing attention [129]. More specifically, the improvement of the technology in recombinant protein production made new penicillin G acylases affordable, which possess enhanced efficiency and stability. As a result of the considerable efforts spent in the optimization of the enzymatic process, 6-APA is currently produced through hydrolysis of penicillin G salt, using water as reagent and immobilized penicillin G acylase as a catalyst [130,131].

**Figure 15:**
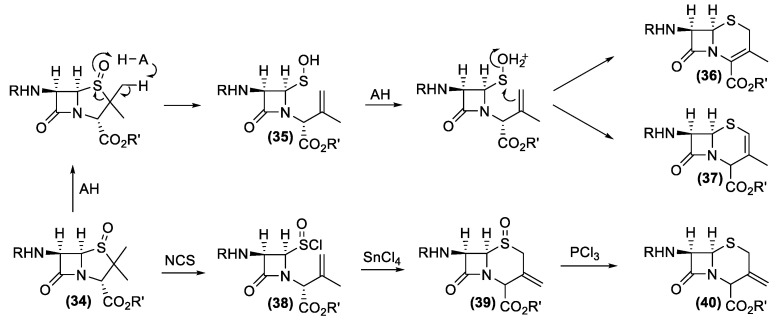
The transformation of penicillin sulfoxides through Morin rearrangement (upper pathway) or through Kukolja rearrangement.

The gained large-scale availability of the 6-APA secured the synthetic access to the main part of the semisynthetic antibiotics possessing the β-lactam nucleus of penicillin. Similarly, the discovery of the cephalosporins produced by *Acremonium* and *Streptomyces* genera fostered a new quest for a suitable synthetic building block for the preparation of semisynthetic cephalosporins. Unfortunately, in those years, the structural complexity of the natural cephalosporins and the lack of high producer strains did not lead to any outstanding fermentative process that could support the synthesis of all the semisynthetic cephalosporins. Consequently, a completely different preparative approach was investigated. More specifically, the research focused on chemical reactions that could transform the easily available penicillin G into new derivatives, possessing the cephalosporin β-lactam nucleus [132]. In this context, two new chemical rearrangement reactions were specially developed (Figure 15). Penicillin sulfoxides (**34**), easily available by oxidation of penicillin, are the starting compounds for both transformations.

The acid-promoted stereospecific transformation of penicillin sulfoxides into corresponding desacetoxycephalosporins (**36**) or (**37**) is generally known as the Morin rearrangement [133]. This reaction is performed by heating sulfoxide (**34**) in presence of an acid catalyst. The process involves a number of steps comprising of activation of the sulfoxide by the acid leading to the generation of the azetidinone sulfenic acid (**35**), protonation of sulfenic acid and cyclization with the adjacent π-bond. The preferential formation of one of the two isomers (**36**) or (**37**) is controlled by the experimental conditions used.

In the same contest, Kukolja discovered the oxidative ring expansion of sulfoxide **34** yielding 3-methylenecepham sulfoxides (**39**) [134]. The reaction is based on the transformation of sulfoxide (**34**) into sulfinyl halide (**38**), by means of halogenating agents. The following cyclization of (**38**) by Lewis acids and the reduction of the obtained sulfoxide (**39**) afford 3-methylenecephams of type (**40**).

Overall, both Morin and Kukolja reactions were widely employed for the industrial synthesis of the cephalosporins [135,136]. As very relevant examples, we report the industrial processes for the synthesis of 7-aminodeacetoxycephalosporanic acid (7-ADCA) and cefaclor.

**Figure 16:**
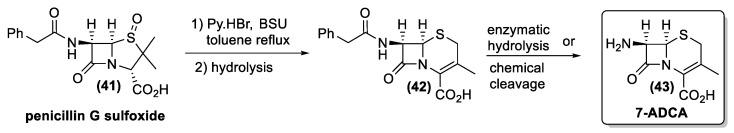
The industrial synthesis of 7-aminodeacetoxycephalosporanic acid (7-ADCA).

Morin rearrangement is the key step in the transformation of penicillin G sulfoxide (**41**) into the cephalosporanic acid derivative (**42)**, whose hydrolysis gives 7-ADCA (**43**), the most important building block for cephalosporin synthesis (Figure 16).

**Figure 17:**
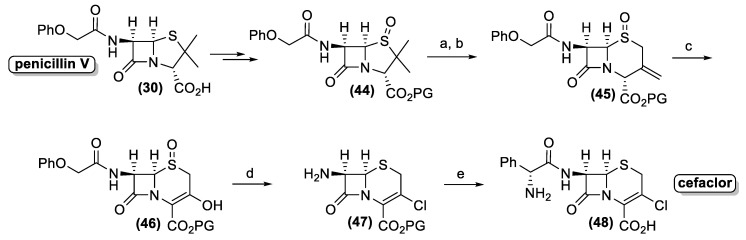
The key synthetic steps of the synthesis of cefaclor^®^ by Eli Lilly Company. PG = protecting group. Reagents and conditions: (**a**) NCS, toluene, reflux; (**b**) SnCl_4_, CH_2_Cl_2_, rt; (**c**) O_3_, CH_2_Cl_2_, −70 °C; (**d**) (PhO)_3_PCl_2_, CH_2_Cl_2_, −15 °C then *i*BuOH; (**e**) 2-amino-2-phenylacetylchloride; deprotection.

Similarly, penicillin V (**30**) is converted into the corresponding protected sulfoxide (**44**), which is transformed into 3-methylenecepham sulfoxides (**45**) by the Kukolja process (Figure 17). The production of the latter pharmaceutical intermediate was exploited in different industrial processes, affording semi-synthetic cephalosporin antibiotic. A representative example is the cefaclor synthesis, developed by the Eli Lilly Company. Accordingly, enol (**46**) is prepared by ozonolysis of compound (**45**).

Hence, the use of the triphenyl phosphite-chlorine complex as a halogenating and reducing reagent allows the one pot transformation of three different functional groups. The halogenation of the enol group affords the corresponding vinyl chloride whereas the sulfoxide group is reduced to sulfide. Finally, the acyl side chain is cleaved through the formation of the corresponding chloro-imine, which is hydrolyzed by addition of isobutanol. The resulting amino derivative (**47**) is treated with phenylglycine chloride followed by the deprotection of the carboxyl functional group to afford cefaclor (**48**).

### 4.2. Tetracyclines

**Figure 18:**
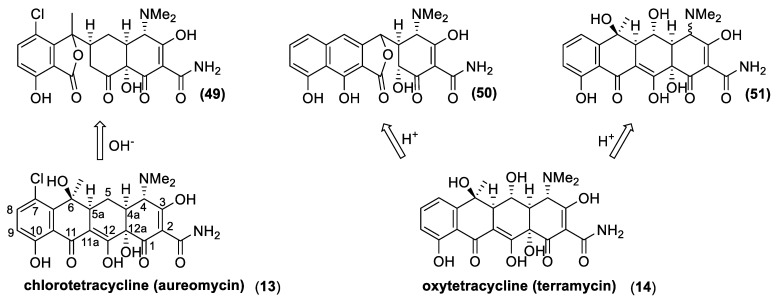
The chemical sensitivity of natural tetracyclines exemplified with aureomycin and terramycin degradation in basic and acidic environments, respectively. Tetracycline numbering is reported for aureomycin.

The history of tetracyclines started in the early 1940s, with the identification of the chlortetracycline (**13**) from a strain of *Streptomyces* isolated from soil [72]. Since the new antibiotics and the *Streptomyces* colonies were gold-colored, the names aureomycin and *Streptomyces aureofaciens* were assigned to the tetracycline, and to the new bacteria species, respectively. The first pharmacological studies revealed that aureomycin is a potent broad-spectrum antibacterial agent, and in 1948, the drug was approved for clinical use, although its exact chemical structure had yet to be determined. Within ten years, three more natural tetracyclines—terramycin (**14**), achromycin (**15**), and declomycin (**16**)—entered the pharmaceutical market. However, the increasing incidence of bacterial resistance to this group of antibiotics led to a decline in their use for human medicine. As a result, the need for the development of new active tetracyclines stimulated the researchers, in their syntheses and synthetic modifications. R.B Woodward, who was awarded the Nobel Prize in Chemistry in 1965, collaborated with Pfizer to determine the structure of terramycin and to study the total synthesis of tetracyclines. Because of the complicated stereochemistry and substitution, Woodward once described these compounds as a “diabolical concatenation of reactive groupings”. Moreover, the many substituents in the tetracyclines render these drugs very sensitive to acidic, alkaline, and reducing reagents; thus, making their stereospecific syntheses an outstanding challenge. As exemplified with aureomycin and terramycin degradation (Figure 18), basic and acidic environments can trigger their degradation [137]. In the presence of bases, the tetracyclines can isomerize to isotetracyclines. Chlortetracycline (**13**) is particularly labile, and forms isochlortetracycline (**49**) even when warmed at pH 7.5. In strong acidic conditions, tetracyclines containing a hydroxyl group at C-6 readily eliminate water with concomitant aromatization of the ring. In these conditions, oxytetracycline (**14**) formed an epimeric mixture of the apo-oxytetracycline (**50**), whereas the epimerization at C-4 occurs readily at pH values between 2 and 6, affording compound (**51**).

**Figure 19:**
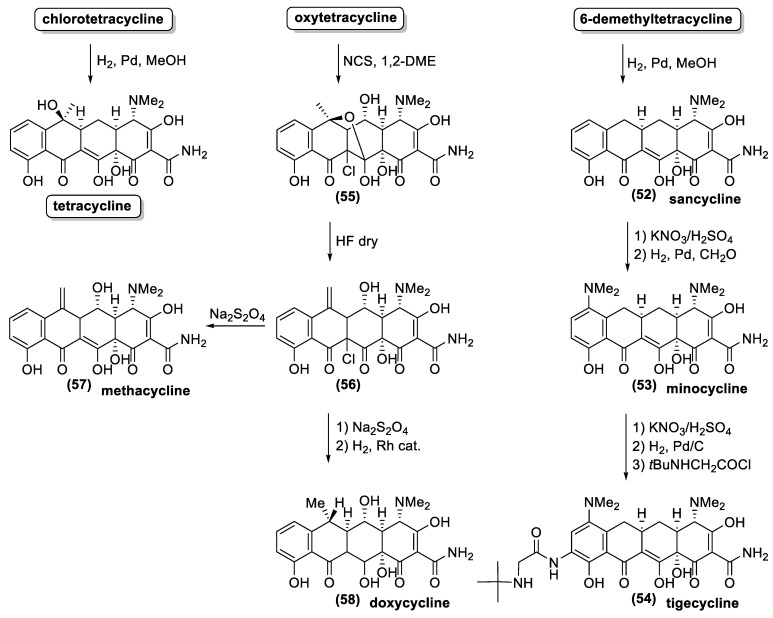
The synthetic approaches to the most relevant semisynthetic tetracyclines.

Due to the above-described synthetic problems, the first enantioselective synthesis of a natural tetracycline was only reported in 2000 [138], almost 60 years after the isolation of aureomycin, the first known tetracycline. On the contrary, the large-scale availability of natural tetracyclines by fermentation fostered the study on the preparation of semisynthetic tetracyclines. Some chemical transformations, suitable for industrial production, were specially developed for these antibiotics [72,125,137,139]. For example, hydrogenolysis of the benzylic hydroxyl groups at C-6 provided a reliable entry into a series of acid-stable 6-deoxytetracycline while the same reaction allowed removing the chloride functional group from the C-7 position (Figure 19). As a result, hydrogenation of the aureomycin and demethylchlortetracycline gave tetracycline and sancycline (**52**). The obtained synthetic tetracyclines showed minor toxicity and higher chemical stability. Moreover, these structural modifications increase the reactivity of the positions 7 and 9 towards electrophilic substitution. Accordingly, the nitration reaction is the key step in the synthetic processes for the preparation of relevant antibiotics, such as minocycline (**53**) and tigecycline (**54**). Another clever procedure for chemical modification of the position 6 is illustrated with the preparation of methacycline (**57**) and doxycycline (**58**) from oxytetracycline. The benzylic hydroxyl group of the latter natural antibiotic is not easily cleaved through hydrogenation because it is quaternary and sterically hindered. Therefore, the addition of chloride, by means of NCS treatment allows the formation of the pentacyclic derivative (**55**). This compound is treated with hydrofluoric acid, and the resulting elimination of one water molecule, affords the key intermediate (**56**). The latter compound possesses an exomethylenic double bond instead of the quaternary hydroxyl group. The reductive removal of the chloride functional group at position 11a gives methacycline (**57**), whereas a further reductive step (by hydrogenation) affords doxycycline (**58**) commercialized under the name Vibramycin^®^.

Overall, the above-described chemical processes provide the main part of the semisynthetic tetracycline on the market. However, the transformation of natural tetracycline allows a limited number of modifications of their chemical frameworks. Otherwise, de novo synthesis would give access to a larger number of new and more different derivatives.

**Figure 20:**
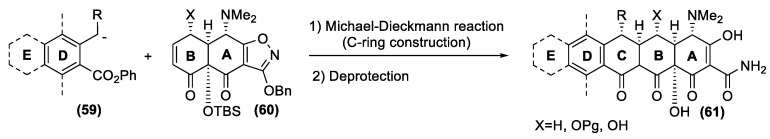
The key steps of the Myers approach to fully synthetic tetracyclines.

As mentioned previously, these drugs are a formidable synthetic challenge, and only in 2005 did the Myers group report a versatile and enantioselective synthetic route to a diverse range of 6-deoxytetracycline antibiotics [140,141]. The new process targeted not a single compound, but a group of structures with the D ring as a site of structural variability (Figure 20). This strategy alternatively involves the parallel preparation of the left fragment (D ring, eventually substituted with a further E ring) and the right fragments (A and B ring) followed by their efficient union, with concomitant formation of the central C ring. The key coupling reaction for merging the two moieties involves a highly stereocontrolled Michael–Claisen cyclization reaction. Accordingly, a carbanion intermediate of type (**59**) undergoes a conjugate addition to the α, β-unsaturated ketone (**60**), followed by ring closure reaction to afford the whole tetracycline framework (**61**). Overall, two carbon-carbon bonds, and two stereogenic centers are created in only one experimental step.

**Figure 21:**
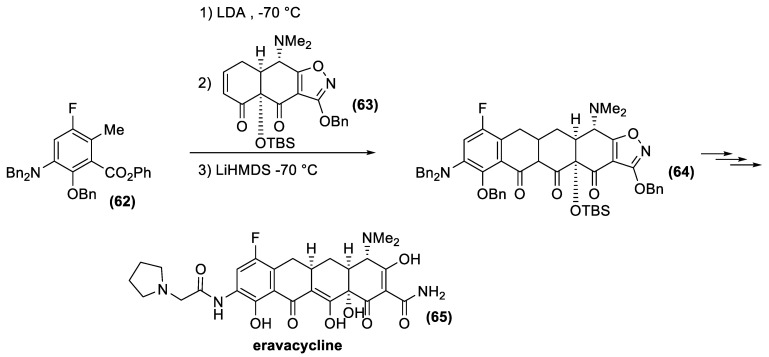
Eravacycline synthesis according to the Myers process.

The extraordinary flexibility of the method has now made it possible to identify new tetracycline derivatives with activity against a wide range of antibiotic-resistant bacteria. In this context, Tetraphase Pharmaceuticals has recently developed eravacycline [142], a synthetic halogenated tetracycline closely related to tigecycline. Eravacycline has shown potent broad-spectrum activity against a wide variety of microorganisms, including many Gram-positive organisms, such as methicillin-resistant *S. aureus*, and vancomycin resistant *Enterococcus faecalis* and *Enterococcus faecium*; Gram-negative organisms, such as *Escherichia coli*, and anaerobic species of microorganisms, such as *Bacteroides*. This fluorocycline was compared to ertapenem and meropenem for the treatment of complicated intra-abdominal infections and levofloxacin for the treatment of complicated urinary tract infections. Eravacycline was granted fast track designation by the FDA and is currently in phase III clinical trials as a broad-spectrum antibiotic. This new, fully synthetic antibiotic is currently produced according to the Myers synthetic protocol (Figure 21). The key building blocks are the fluoro-benzoate (**62**) and the α, β-unsaturated ketone (**63**), whose condensation affords tetracycline derivative (**64**). Finally, deprotection reactions and functional group transformation give eravacycline (**65**).

### 4.3. Lipiarmycins

*Actinomycetes* are producers of different antibiotics, whose chemical structures range from relatively small β-lactam derivatives to the larger macrolide derivatives. All of these substances have high structural complexity in common, which represents an exceptional challenge concerning both structure determination and synthesis. Of course, a higher molecular complexity is linked to tricky analytical procedures and synthetic processes.

The histories of the most important antibiotics are very similar to each other. Usually, their studies started many years ago, with the isolation from a given Actinomyces strain followed by the struggle for the determination of their chemical structures. Meanwhile, the study of their total syntheses helped to confirm the structure assignation and laid the foundations for the preparation of new semi-synthetic or fully synthetic analogues. Although, with respect to their specific features, the histories of a number of macrolide antibiotics, such as erythromycin, vancomycin, rifamycin, daptomycin, and their semisynthetic analogues have followed the above-described path.

**Figure 22:**
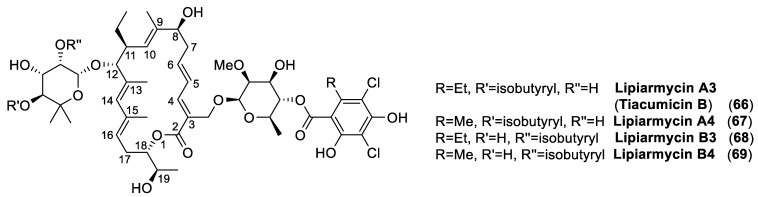
The chemical structures of lipiarmycins.

In order to illustrate a very relevant example, we singled out the less known antibiotic lipiarmycin A3 (Figure 22). The reason for our choice lies in the long and intriguing history of this natural product. Indeed, this macrolide returned to the fore as demonstrated by the great number of scientific studies that were published in very recent years [143,144,145].

Lipiarmycin was first isolated in the early seventies by the Lepetit group (Italy), during a screening for antibiotics produced by strains of the genus *Actinoplanes*. A new *Actinoplanes* specie [146] was identified from a soil specimen collected in locality Decca in India and, accordingly, the bacterium was named *Actinoplanes deccanensis*. This microorganism produced a substance with strong activity against Gram-positive bacteria. The new antibiotic was isolated on February 29, 1972, and was named lipiarmycin (from leap year).

Albeit, its chemical physical properties and its biological activity [147,148] were thoroughly described within a few years later, this material was recognized to be a mixture of two related products in 1987, when the first comprehensive study [149] on the chemical structure determination of lipiarmycin was published. On the basis of chemical degradations and NMR studies, the antibiotics extracted from *Actinoplanes deccanensis*, named lipiarmycin A3 (**66**) and lipiarmycin A4 (**67**), were characterized by a common 18-membered macro lactone attached to two glycosyl moieties, namely 2-O-methyl-4-O-homodichloro-orsellinate-β-D-rhamnose and 4-O-isobutyrate-5-methyl-β-rhamnose (Figure 22). Further studies [150] revealed that the same strain was also able to produce two minor compounds, lipiarmycin B3 (**68**) and B4 (**69**), respectively, which differed from the corresponding A3 and A4 by the position of the isobutyric ester on the rhamnose moiety.

In the meantime, two research groups from Japan United States independently reported the isolation of clostomicins, from *Micromonospora echinospora* subsp. *armeniaca* [151], and tiacumicins from *Dactylosporangium aurantiacum* subsp. *hamdenensis* [152], respectively. Based on NMR studies, clostomicin B1 and tiacumicin B were recognized as identical to lipiarmycin A3. In the same years (The late 80s), a very large number of effective semisynthetic antibiotics with broad activities were developed and the studies on this small class of compounds were interrupted for more than a decade.

A renewed interest in lipiarmycin A3/tiacumicin B started in the late 1990s, when Optimer Pharmaceuticals began the commercial development of tiacumicin for the treatment of *Clostridium difficile* [153,154]. This Gram-positive bacterium is an important nosocomial pathogen frequently diagnosed in infectious hospital-acquired diarrhea; it is regarded as a major public health concern. Tiacumicin B (Fidaxomicin) was then approved by the FDA in 2011; it became the first-line therapy for the cure of *Clostridium difficile* infections.

Increasing interest in tiacumicin B induced more detailed structural studies, culminating in the determination of its structure by single crystal X-ray analysis [155]. The stereo structure **66**, possessing the (18*S*,19*R*) absolute configuration, was thus assessed for tiacumicin B. In this context, the (19*S*)-diastereoisomer of **66** was prepared by synthesis and the spectroscopic analyses of the latter compound were not compared to those of tiacumicin B, but appeared identical to those reported for lipiarmycin A4 (**67**). However, the NMR spectra of the semi-synthetic and of the tiacumicin B (of unambiguously determined configuration) were acquired in different solvents, thus inhibiting the evaluation of the subtle spectroscopic differences between the two. Following the same reasoning, lipiarmycin A4 was assigned the (19*S*) configuration without any further confirmatory analysis.

According to the pharmaceutical regulations adopted by the majority of the nations, even a little difference in the chemical structures of two pharmaceutical products imply the need to present a new common technical document (CTD) for the new substance. Therefore, the determination of the lipiarmycin A3 and tiacumicin B structures and/or the demonstration of their co-identity became a relevant issue for the pharma industry [143]. In addition, recent studies [156] demonstrated that lipiarmycin possessed good activity against multidrug-resistant strains of *Mycobacterium tuberculosis*, expanding its pharmaceutical relevance.

**Figure 23:**
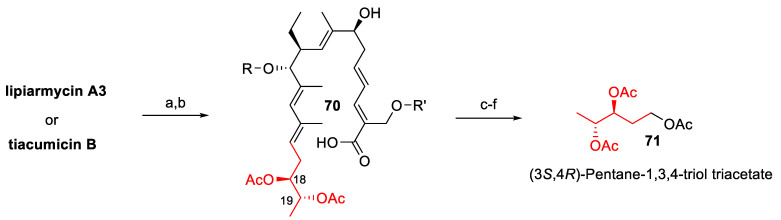
The chemical degradation of lipiarmycin A3 and tiacumicin B allows assigning (18*S*,19*R*) absolute configuration. Reagents and conditions: (**a**) KOH aq.; (**b**) Ac2O, Py; (**c**) O3, MeOH/CH2Cl2, −78 °C; (**d**) NaBH4, from −78 °C to rt; (**e**) chromatographic separation; (**f**) Ac2O, Py, DMAP.

For these reasons, in the last few years, a number of research groups have published studies on the determination of the lipiarmycin/tiacumicin structure, as well as on its total synthesis. More specifically, the chemical degradation of samples of tiacumicin B from *Dactylosporangium aurantiacum* and lipiarmycin A3 from *Actinoplanes* allowed the isolation of the same derivative **71** (Figure 23), which proved identical to a synthetic sample of **71** and unambiguously demonstrated that lipiarmycin A3 and tiacumicin B possess the same (18*S*,19*R*) absolute configuration (2014) [157].

One year later (2015), three different groups independently reported [158,159,160] the enantioselective synthesis of the tiacumicin B aglycon and of the putative lipiarmycin aglycon, followed by the first total synthesis of the fidaxomicin [161]. The latter synthetic compound proved identical to a commercial sample of tiacumicin B from *Dactylosporangium aurantiacum*, thus confirming the structure assigned. Finally, a study on the comparative X-ray analysis of lipiarmycin A3 and tiacumicin B was published in 2017, providing the definitive proof that these pharmaceutical compounds are identical in all respects and confirmed the previous assigned chemical structure of lipiarmycin A3 [162].

Although the debate on the lipiarmycin structural determination is now closed, the studies on the synthesis of this antibiotic have increased, fostered by renewed interest for the preparation of synthetic derivatives. Accordingly, two new syntheses of tiacumicin B aglycone [163,164], the total synthesis of tiacumicin A [165], the total synthesis of tiacumicin B [166], and two reviews on tiacumicin B [144,145] were reported between 2017 and 2020.

**Figure 24:**
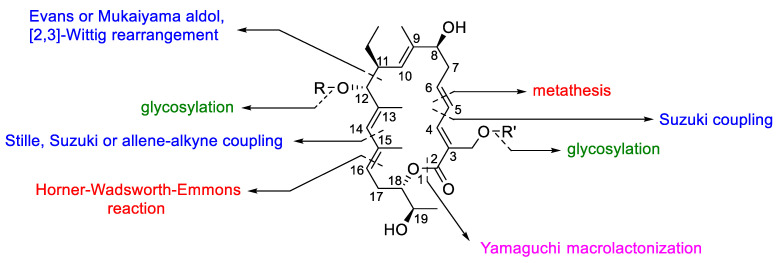
The key synthetic steps employed in the synthesis of lipiarmycins. The reaction used to build the indicated carbon–carbon single bonds, carbon–carbon double bonds, macrolactone bond, and glycosidic bonds are highlighted with blue, red, pink, and green color, respectively.

Although the synthetic strategies are very similar each other, the order of assembling the fragments change between the different approaches (Figure 24). For example, the final ring closure of the lactonic ring was built-up either applying a ring closing metathesis between C(5) and C(6) or through Yamaguchi macrolactonization. The Suzuki, Stille, or the allene–alkyne coupling were employed for the formation of the carbon-carbon single bond between C(4) and C(5) and between C(14) and C(15). In addition, the carbon–carbon single bond between C(11) and C(12) was formed through aldol based reactions or [2,3] the Wittig rearrangement, whereas the carbon–carbon double bond between C(15) and C(16) was obtained by Horner–Wadsworth–Emmons reaction.

Overall, none of the reported synthetic approaches were designed in order to secure an industrial process for lipiarmycin preparation. Nevertheless, they represent a relevant advance in the study of this antibiotic, providing new chemical insights for the design of new semisynthetic derivatives [167,168].

## 5. *Actinomycetes* Are still Considered a Source of Bioactive Compounds

It seems that regarding antibiotics all has been already discovered. Despite this common perception, *Actinomycetes* remain the most important source of genetic variability in different ecosystems. Molecular biology and genetics give us new equipment that permit better sensitivity and accuracy in finding new specific microbial molecular targets and bioactive compounds. Screening techniques become more sophisticated and precise and are based essentially on (i) the use of antisense RNA; (ii) new adjuvant-molecules; (iii) new natural antibiotic-resistant bacteria; (iv) new cultivation methods; and (v) activation of silent operons responding to the secondary metabolism [169].

The antisense RNA is a particular small single-stranded RNA able to pair with a specific mRNA of the cell, causing its degradation and, consequently, the missed translation of the cell genetic heritage. Thanks to this knowledge, we are now able to build specific vectors carrying the information to synthetize these small antisense RNAs inside the cell in order to impair the expression of a specific target gene. This technique permits us to choose new possible target(s) for the therapy, observing altered bacterial phenotypes in terms of morphological changes, impaired growth, and toxicity. Moreover, we are able to validate the mechanism of action of a molecule against the presumed target, enhancing our sensibility in screening protocols [170]. It is possible to try to restore the sensitivity to a drug using an adjuvant molecule. This goal could be reached creating a screening system, seeding a specific antibiotic resistant strain in a plate containing the same antibiotic at which it is resistant. At this point, a molecule or an extract of a fermentative broth could be added, evaluating the restored antibiotic toxicity.

Finally, it is possible to study the target and understand the mechanism of action. Natural antibiotic-resistant bacteria help our new drug research because we start from the premise that natural resistant bacteria could be produce the same antibiotic at which it is resistant (first filter). A second cut-off, based on the known antibiotic open reading frames (ORFs), is then applied, enriching our microbial library in probable new drug producers [171]. Our cultivation procedures are not suitable to permit the growth of a big portion of the prokaryotic world [172]. Enhancing cultivation technologies and comprehending the molecular biology of bacteria could give access to a major number of possibilities to study the biodiversity and the enzymatic tools in the environment [173,174]. This is possible, for example, using strong selective factors, i.e., higher salinity, different temperatures, alkaline or acid growth media (collections of novel or poorly represented strains). Moreover, it is possible to recreate the microenvironment in an in vitro scale, to understand the “cross-talk molecules” implied in the relationship among the bacterial populations.

Another relevant aspect concerns the syntheses of new semisynthetic antibiotics. *Actinomycetes* are producers of bioactive compounds belonging to each one of the most representative classes of antibiotics. These APIs are currently produced through well-established, high volume fermentation processes. Therefore, the pharmaceutical industry can take advantage of these useful building blocks for the creation of new APIs. The development of antibiotic-resistant bacteria leads to the need for new antibiotics and the chemical modification of the known ones is still the most successful approach adopted to this end. To date, the main part of the antibiotics used in clinical practice are semisynthetic derivatives of compounds obtained through *Actinomycetes* strain fermentation. Consequently, understanding the mechanisms of antibiotic resistance can also help design the chemical modification of a given API in order to obtain a new compound with higher biological activity. On the other hand, chemical research will provide the synthetic and technological tools for the industrial production of new antibiotics.

## 6. Conclusions

Bacteria evolutionary success depends on their capability to adapt themselves quickly to an environmental stress, thanks to the fine-tuning of their mutational rates [175]. The mutation could establish (or not) in the population, based on its fitness advantage and, of course, a general evolutionary advantage. Obviously, the mutational rate depends on the growth speed of the population: the faster they replicate, the more mutational events could occur.

Moreover, the role of the toxin–antitoxin system in bacterial persistence was underlined. The activation of these loci permits bacteria to arrest their growth under different stress pressures, such as starvation, non-optimal pH, temperature, high population density, or antibiotic presence in the environment [176,177].

Whatever the mechanism is, the result is the temporary inhibition of the essential cellular processes, such as DNA replication, transcription, and translation. The most important antibiotics perform their actions against active and proliferating cells and they have no effect on these quiescent cells.

It seems clear that their capability to adapt to a stress is a big issue to overcome. The discovery of new useful, clinical drugs, is slow, for sure, and particular attention must be kept using antibiotics. Researches of new microbial targets and new high-performance drugs must go forward. *Actinomycetes*, with their extraordinary metabolisms and their enzymatic and genomic potential, could help us in this aim, providing bioactive compounds never observed or unique structural moieties that chemists could modify in order to obtain other useful compounds. Moreover, the study of their biology and metabolism could assist not only medicine, but also agriculture, cosmetics, laundry, pharmaceutics, and bioremediation [178]. It also important that different scientific fields share their experience, knowledge, technologies, and expertise to improve health and wellness.

“Somewhere something incredible is waiting to be known” (Carl Sagan, astronomer).

## Figures and Tables

**Table 1:** Natural β-lactam antibiotics and β-lactamase inhibitors produced by *Actinomycetes*.

β-Lactams		Producer Microorganism ^1,2^
**Penicillin**	Penicillin N	*S. lipmanii*; *S. microflavus* [38,39]; *S. griseus* subsp. *Cryophilus* [46]; *S. cattleya* [48]
**Cephalosporines**	Cephamycins	*S. clavuligerus* [38,39]; *S. griseus*; *S. lactamdurans* [41]; *S. chartreusis* [42,43,44]; *S. cinnamonensis*; *S. fimbriatus*; *S. halstedii*; *S. rochei*; *S. viridochromogenes* [41]; *S. wadayamensis*; *S.* *Jumonjineneis*; *S. lipmanii*; *S. panayaensis*; *Amycolatopsis lactamdurans* [45]; *S. cattleya* [48]; *S. heteromorphus* [49]
	Cephalosporines	*S. microflavus*; *S. lipmanii*; *S. clavuligerus* [38,39,40,42]; *S. hygroscopicus* [43]
**Carbapenems**	Thienamycins	*S. cattleya* [45,47]; *S. griseus* subsp. *Cryophilus* [46,50]; *S.* *penemifaciens* [48]; *S. flavogriseus*; *S. olivaceus*; *S. cremeus* subsp. *auratilis*; *S. flavoviridis* [49]
**Monobactam**	Nocardicins	*Nocardia uniformis* [45]; *Nocardia uniformis* subsp. *Tsuyamanensis*; *Streptomyces alcalophilus* sp. nov. [49]; *Actinosynnema mirum* [48,49]; *Microtetraspora caesia* [51]
**PBP inhibitors**	Clavulanic acid	*S. clavuligerus*; *S. jumonjinesis*; *S. katsuharamanus* [45]
	Olivanic acid	*S. olivaceus* [46]; *Streptomyces griseus* subsp. *cryophilus* [49]

^1^*S*. indicates the genus *Streptomyces*; ^2^ the numbers in square brackets indicate literature references.

**Table 2:** Natural tetracycline antibiotics produced by *Actinomycetes*.

Tetracycline Antibiotics	Producer Microorganism ^1,2^
**Tetracycline**	*S. aureofaciens*; *S. avellaneus*; *S. lusitanus*; *S.viridifaciens* [38,39,74,75]
**Chlortetracycline**	*S. lusitanus*; *S. aureofaciens*; *S. lividans*^3^ [38,39,74,75,76,77]
**Oxytetracycline**	*S. alboflavus*; *Streptomyces albofaciens*; *Streptomyces erumpens*; *S. griseus*; *S. platensis*; *S. rimosus* subsp. *rimosus*; *S. varsoviensis* [38,39,74]
**6-demethyltetracycline**	*S. aureofaciens*^3^ [77]

^1^*S*. indicates the genus *Streptomyces*; ^2^ the numbers in square brackets indicate literature references ^3^ genetically modified strain.

**Table 3:** Natural aminoglycoside antibiotics produced by *Actinomycetes*.

Aminoglycoside Antibiotics	Producer Microorganism ^1,2^
**Streptomycin**	*S. griseus*; *S. bikiniensis*; *S. streptomycinii*; *S. ornatus*; *S. humidus*; *S. subrutilus*; *S. mashuensis*; *S. glaucescens*; *S. griseocarneus*; *Streptomyces rimosus* subsp. *rimosus*; *S. galbus* [38,39,53,85,86,87]
**Neomycin**	*S. fradiae*; *S. catenulae*; *S. chrestomyceticus*; *S. albogriseolus* [38,39,53,85,88,91]
**Tobramycin**	*Streptoalloteichus hindustanus*; *S. tenebrarius*; *S. cremeus* [38,39,53,88,89]
**Kanamycin**	*S. kanamyceticus* [38,39,53]
**Paromomycym**	*S. rimosus var. paromomycinus*; *S. catenulae*; *S. chrestomyceticus*; *S. rimosus*; *S. fradiae* [38,39,53,85]
**Gentamycin**	*Micromonospora purpurea*; *Micromonospora pallida*; *micromonospora echinospora* [38,39,53,85,90]

^1^*S*. indicates the genus *Streptomyces*; ^2^ the numbers in square brackets indicate literature references.

**Table 4:** Natural peptide antibiotics produced by *Actinomycetes*.

Peptide Antibiotics		Producer Microorganism ^1,2^
**Glycopeptides**	vancomycin	*Amycolatopsis orientalis* subsp. *Orientalis* [38,39]
**Lipopeptides**	daptomycin	*Streptomyces* sp. *KCTC12267BP* [105]; *S. roseosporus* [106,108]; *S. lividans* ^3^ [107]
**Streptogramins**	Streptogramins A and B	*S. halstedii* [38]; *S. pristinaespiralis*; *S. virginiae* [109]

^1^ S. indicates the genus *Streptomyces*; ^2^ the numbers in square brackets indicate literature references; ^3^ *S. lividans* genetically modified strain.

## Data Availability

No new data were created or analyzed in this study.
